# Delayed Presentation of Osmotic Demyelination Syndrome Treated With Plasmapheresis

**DOI:** 10.7759/cureus.47399

**Published:** 2023-10-20

**Authors:** Muhammad Waqar Sharif, Arjan Singh, Joud Enabi, Roman Karkee, Raghavendra Sanivarapu

**Affiliations:** 1 Internal Medicine, Texas Tech University Health Sciences Center, Odessa, USA; 2 Pulmonary and Critical Care Medicine, Permian Basin Campus, Texas Tech University Health Sciences Center, Midland, USA

**Keywords:** nosocomial aspiration pneumonia, therapeutic plasmapheresis, central pontine myelinolysis (cpm), treatment of hyponatremia, alcohol use disorder (aud)

## Abstract

We present a unique case of a 42-year-old gentleman with alcohol use disorder who developed osmotic demyelination syndrome (ODS) despite appropriate hyponatremia correction. This patient initially presented with severe hyponatremia (Na 97 mEq/L) due to beer potomania, which was corrected gradually over eight days, resulting in no observed neurological deficits upon discharge. However, he was readmitted with respiratory failure from aspiration pneumonia, leading to endotracheal intubation. Laboratory findings revealed a sodium level of 134 mEq/L and serum osmolality (293 mOsm/kg). The patient had neurological exam findings of spontaneous eye opening with left gaze preference and decreased power ⅕ in all extremities. Following extubation, he experienced a relapse with evolving subacute central pontine myelinolysis and bulbar weakness necessitating reintubation. Subsequently, five sessions of plasmapheresis were conducted, resulting in stable clinical findings. Despite remaining non-verbal, the patient demonstrated gradual neurological motor improvement, progressing from 1/5 power in all extremities to 4/5 on the right side and 3/5 on the left side. He was discharged with ventilator support, tracheostomy, and PEG tube placement to a long-term care facility. This case underscores the importance of vigilant monitoring in high-risk individuals following hyponatremia treatment because ODS presentation can be delayed.

## Introduction

Osmotic demyelination syndrome (ODS) is a neurological disorder characterized by the non-inflammatory demyelination of specific areas within the central nervous system, particularly the pons, and sometimes involving extrapontine regions [1]. However, ODS is still considered to be rare in adults, with an incidence of 0.3-1.1% of hospital admissions​ [2]. Rapid correction of hyponatremia is the most common etiology of ODS in up to 80% of cases especially when the correction exceeds 12 mmol/L within a 24-hour period​ [3]. Although imbalances in sodium levels are the most common cause of ODS, imbalances of nearly all other electrolytes, like phosphorus and potassium, may lead to CNS demyelination as well​ [3]. Some groups of patients are at higher risk of developing this syndrome, including patients with hepatic failure, kidney failure, and alcoholics​ [4]. Additionally, diabetes mellitus, particularly during diabetic ketoacidosis or hyperosmolar hyperglycemia episodes, can elevate susceptibility to ODS, particularly when combined with significant osmolality changes [3]. The classical clinical manifestations of ODS follow a biphasic course with mild encephalopathy initially due to the electrolyte imbalance that improves after the electrolyte correction, followed by neurological deterioration with dysphagia, dysarthria, and variable degrees of quadriplegia​. It takes 1-14 days for the manifestations of ODS to present​ [3]. Brain magnetic resonance imaging (MRI) is the diagnostic modality of choice. T2-weighted and fluid-attenuated inversion recovery (FLAIR) shows hyperintensity and hypointensity in T1-weighted sequences in the central pons with a characteristic trident appearance with sparing of the tegmentum and ventrolateral pons [5,6]. Differentials for the imaging findings include multiple sclerosis, multi-infarct dementia, and encephalitis, which produce areas of increased T2 signal in the pons that resemble those of central pontine myelinolysis, but these conditions also cause significant periventricular abnormalities and a distinctive clinical picture [7]. To prevent ODS when treating chronic hyponatremia, certain guidelines for sodium correction have been proposed. According to the American Expert Panel, for individuals at an average risk of ODS, the recommended limit for sodium correction is 10-12 mEq/L within 24 hours. High-risk patients should not exceed 8 mEq/L of correction within the first 24 hours [8]. The primary approach to treating myelinolysis is mainly centered around providing supportive care. However, there have been recent reports suggesting that therapeutic plasmapheresis can be a reliable and safe method to enhance the patient's overall clinical condition. Here, we present a case of a 42-year-old gentleman with alcohol use disorder who presented to our center with a three-day history of generalized weakness, confusion, extremities weakness, and intermittent choking episodes. Fourteen days before this presentation, he was admitted for severe hyponatremia (Na 97 mEq/L) secondary to beer potomania. 

## Case presentation

The patient is a 42-year-old male with a medical history significant for chronic alcohol use disorder started drinking in his twenties, 3-4 cans of beer every day with increased in frequency to 10 cans every day in the last four days. Additionally, he has a 15-pack-year smoking history with ongoing tobacco use, a three-year history of well-controlled hypertension on 100mg of losartan, and mild asthma on as-needed albuterol. He was sent to our hospital by his primary care physician (PCP) due to significantly low sodium levels. The patient was being evaluated by his PCP for complaints of dizziness and weakness. Approximately 15 days before coming to the Emergency Department (ED), his sodium levels were 115 mEq/L, and repeat sodium was 110 mEq/L in the clinic; thus, he was sent to our hospital. On arrival at the ED, he complained of burning epigastric pain and intermittent productive cough but denied any other symptoms. In the ED on Day 0, his sodium levels were 97mEq/L, serum osmolarity 208 mOsm/kg, urine osmolality 90 mOsm/kg, and urine sodium < 5 mmol/L. He was admitted with a working diagnosis of severe hypotonic euvolemic hyponatremia secondary to beer potomania and treated with fluid restriction and tolvaptan. The hospital course was complicated by fluid overload; therefore, he was effectively diuresed with furosemide 40mg daily until he was on room air. The rate of correction of sodium was 8-10 meq/24 hours. He was discharged home on salt tablets after gradually correcting sodium to 131 mEq/L in 8 days and serum osmolality of 270 mOsm/kg. Other home medications on discharge included chlordiazepoxide 10 mg TID, folic acid 1 mg daily, furosemide 40 mg oral tablet PO daily, and vitamin B1 100 mg oral tablet TID. No neurological deficits were present at the time of discharge. 

**Figure 1:**
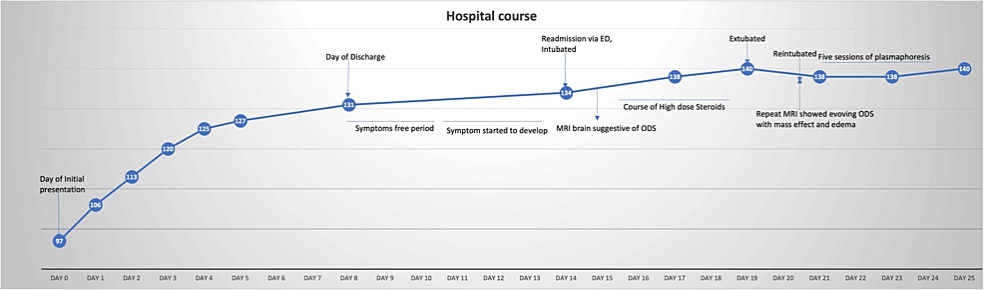
Hospital course with arrows pointing toward the event with sodium levels on the line graph (mEq/L) and number of days on the X-axis

After hospital discharge, he presented again with a three-day history of generalized weakness prominent over bilateral lower extremities and altered mental status with increasing confusion. Shortly following discharge, he started developing non-bloody diarrhea 2-3 times/day, became increasingly weak, and had multiple falls associated with minor injuries. The cause of diarrhea remained unknown as the gastroenteritis panel for bacteria and virus was negative. Multiple episodes of choking on liquids and persistent productive cough were also associated with this presentation. This time, he was intubated in the ED and admitted to the ICU for respiratory failure secondary to aspiration pneumonia. The hospital course is shown in Figure 1. 

During the second admission on day 14. He was febrile, 103 F, tachycardic 123/m, and the rest of the vitals were stable. On auscultation of the lungs, coarse breath sounds were heard on bilateral lung fields; the neurological exam was unreliable due to sedation. Sodium was 134 mmol/L, and serum osmolality was 293 mOsm/kg. CT Brain w/o contrast was done in the ED to rule out a brain bleed which came out to be a normal exam except for chronic left occipital scalp hematoma. 

**Figure 2:**
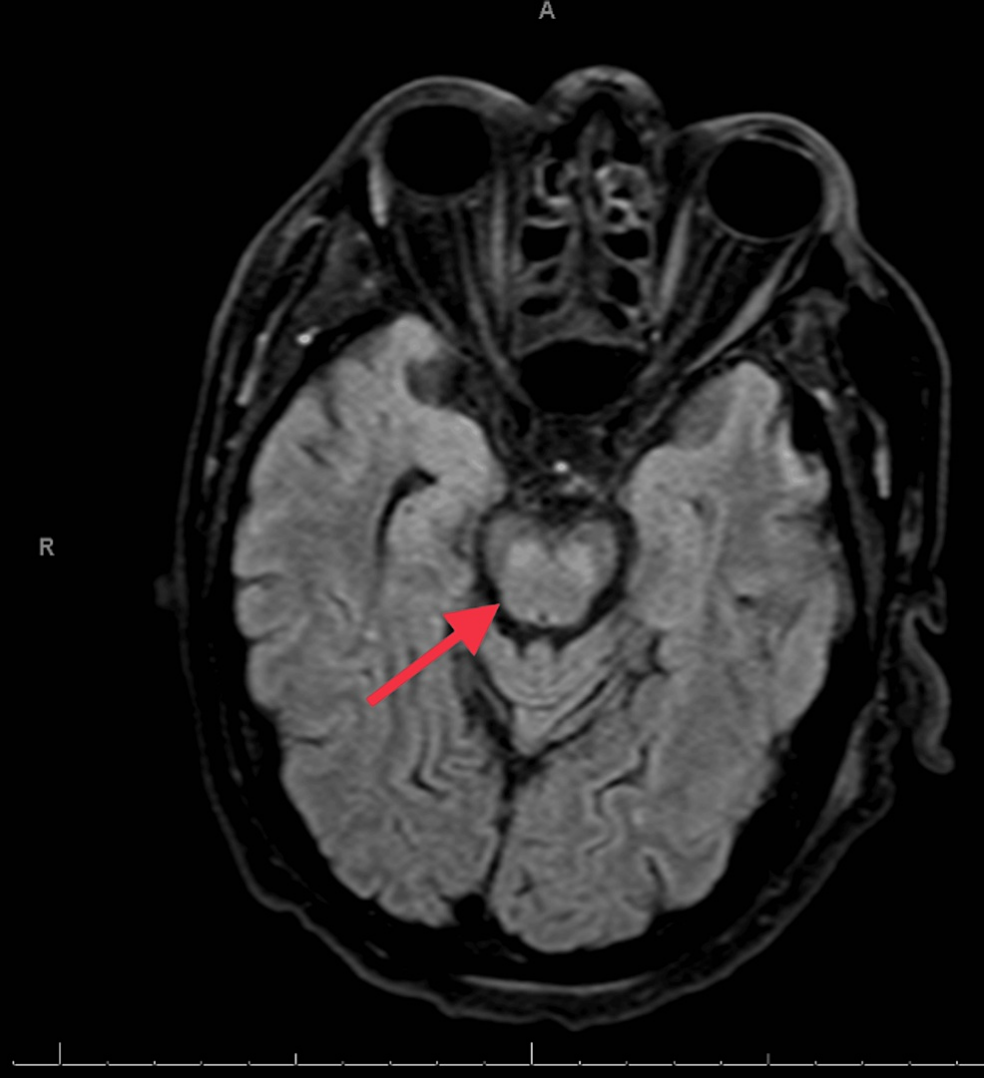
MRI T1 view coronal section showing acute central pontine myelinolysis

On day 15, the neurological exam was done off sedation (propofol) for 30 mins; the patient remained obtunded, was not following commands, had miotic pupils, and was minimally reactive midline, corneal intact B/L, cough, and gag reflex intact. No motor response to pain was seen in arms b/l. Legs showed slight movement in response to pain. He did moan in response to pain stimulation in his legs. MRI brain w/o contrast revealed acute central pontine myelinolysis/osmotic demyelination syndrome, as shown in Figure 2. These MRI findings were correlated with the neurological findings. 

Neurology was consulted once a brain MRI confirmed ODS. He was started on 1g IV methylprednisolone daily for five days, along with tight control of Na between 130 and 140 mmol/L. The patient was extubated following improvement of respiratory function. However, two days later, he had to be reintubated because of an aspiration event related to bulbar weakness. At this time, a repeat MRI of the brain revealed evolving subacute central pontine myelinolysis with mass effect. Following this, plasmapheresis was initiated on day 24, and five sessions of plasmapheresis were completed under the guidance of the nephrology service. Subsequent brain imaging showed stable findings.

Differential diagnosis

During the second admission, the patient presented with severe weakness, multiple falls, and a history of choking episodes. Given the recent history of treatment for severe hyponatremia, we suspected osmotic demyelination syndrome, which was later confirmed by brain imaging. Guillain-Barre syndrome was among the initial differentials, considering the history of recent diarrhea associated with prominent lower extremity weakness and associated respiratory difficulty +/- choking episodes. However, the rapid onset of symptoms and lack of an ascending pattern of weakness deemed GBS less likely. Alcohol-induced peripheral neuropathy was a differential but could not explain the acute onset of the weakness. Other differentials that were excluded based on imaging and history were acute flare of multiple sclerosis, pontine infarction from basilar perforators, although usually, brainstem infarcts stop at the midline, pontine neoplasms including astrocytomas, brainstem metastasis, and vigabatrin toxicity which would be seen as bilateral dorsal pontine longitudinal restricted diffusion on MRI [6]. Hence based on history, physical exam, and imaging findings, it was established that our patient had delayed presentation of ODS. 

Outcome and follow-up

Clinically, the patient remained non-verbal but showed serial improvement in neurological motor function, from 1/5 power in all extremities to 4/5 on the right side and 3/5 on the left side extremities. Due to associated bulbar weakness, the patient was ventilator-dependent, requiring tracheostomy and PEG tube placement. Eventually, he was discharged to a long-term facility. Three months after discharge, the patient continued working with physical, speech, and language therapy. Substantial recovery of his neurological function was noted. His verbal skills had improved, and his dysarthria was getting better. He could drink liquids without choking or coughing and could eat a mixture of liquid and solid foods. He was also able to walk without assistance at a moderate pace.

## Discussion

ODS is a rare and life-threatening condition with an estimated prevalence of 0.25% to 0.5% in the general population​ [1]. Although the prognosis has improved with the advancement in diagnostic imaging and management, 33% to 55% of patients still die or require some assistance in daily life​ [9]. Recent studies show that hyponatremia remains the most important cause of ODS, with severe hyponatremia (serum sodium < 120 meq/L) present in 47% of cases ​[9]. While rapid correction of sodium (>10 meq/L/day) is a known trigger for ODS, other comorbidities like chronic alcoholism (seen in 40% of patients), as seen in our patients, should be considered in times of uncertainty [1,9]. ODS can arise during the last stage of binge drinking in patients with alcohol use disorder due to the osmotic changes resulting from reduced food and water intake during these times. This mechanism probably describes one of the possible ways our patient developed ODS [10]. 

Clinical manifestation depends on the site of involvement: central pontine myelinolysis (CPM), extrapontine myelinolysis (EPM), or mixed, with typical symptoms appearing 1 - 14 days after the initial insult​ [11]. Common clinical manifestations in CPM, as seen in our patient, include quadriparesis and bulbar weakness, which have been reported in 9.8-28.8% and 3.2 -11.5% cases, respectively. However, it is important to note that the delayed presentation of ODS has been sparsely mentioned in the literature. We highlight that our case exhibited a period of latency after optimal sodium correction, developing typical features of ODS almost 14 days later.

The pathogenesis of ODS involves oligodendrocyte shrinkage and demyelination. In the setting of chronic hyponatremia, neuronal cells, oligodendrocytes, in particular, release osmolytes into extracellular space to prevent cell swelling. With rapid sodium correction in such circumstances, neuronal cells cannot regenerate osmolytes fast enough, eventually resulting in cell shrinkage. Also, osmotic stress induces the release of myelotoxic agents from neuronal cells, which results in ongoing oligodendrocyte injury and demyelination, eventually precipitating ODS​ [12]. 

The importance of discussing the prevention of ODS comes from the fact that there is a paucity of studies on epidemiology and standardized treatment guidelines. In several reported cases, plasmapheresis, steroids, and IVIG used alone or in combinations have shown benefits in ODS, although the outcomes were variable [12-14]. The rationale of using plasmapheresis in our case is that it reduces these myelotoxic agents, which in turn prevents ongoing neuronal insult. In multiple studies, outcomes from plasmapheresis were variable, ranging from no recovery to partial or complete recovery.

In our case, although plasmapheresis was initiated on the tenth day of symptom onset, the patient responded well, with complete neurological recovery after a total of six cycles. Therefore, with this case report, we try to serve two purposes: first, explain the variable nature of ODS presentation and the need for physicians to be familiar with its risk groups and clinical presentation; second, highlight that it is justifiable to start plasmapheresis in patients with ODS, regardless of the duration of symptoms onset as Table 1 shows a great comparison between patients with different clinical features, variation in onset of plasmapheresis and neurological outcomes. The direct effects of plasmapheresis on the blood-brain barrier have not been proven, although improvement in neurological symptoms can be explained by the removal of myelinotoxic substances, which are involved in the pathogenesis of ODS [13].

**Table 1:** Cases of delayed presentation of ODS treated with plasmapheresis reported in the literature

Patient Particular	Risk Factors	ODS variant (CPM, EPM, or mixed)	Clinical feature	Plasmapheresis initiation (Days after symptoms onset)	Number of cycles/ volumes replaced	Neurological Outcome
23 Y/M​ [15]​	Severe Hyponatremia Rapid Sodium correction Hepatitis B	CPM	Unresponsive to commands, spontaneous eye opening and withdrawal to pain	14^th^ day	4 cycles 15,500 ml	Partial recovery – follows commands Tracheostomy dependent
71 Y/ F​ [16]	Severe hyponatremia Rapid sodium correction	Mixed	Bulbar weakness with Progressive dysarthria and gait disorder progressing to comatose state	39^th^ day	6 sessions	Complete Recovery
43 Y/M​ [14]​	Severe Hyponatremia Diabetes Mellitus II	Mixed	Reduced level of consciousness, Parkinsonism (bradykinesia and tremors of both upper and lower limbs symmetrically)	N/A	5 cycles	Complete Recovery
40 Y/ F​ [12]	Hypokalemia Intravenous Sodium Bicarbonate Therapy	CPM	Tetraparesis, Bulbar weakness	6 days	2 cycles 4394 ml	Partial Recovery with residual diplopia
63 Y/M [17]	Severe Hyponatremia Rapid correction of sodium	CPM	Status epilepticus with facial twitching	6 days	N/A	Residual left hemiparesis and sensory ataxia, assisted ambulation with a walking stick
50 Y/F​ [18]​	Severe hyponatremia Chronic Kidney Disease	N/A	Bulbar weakness – dysarthria Rigidity and tetraparesis	21 days	7 cycles	Complete recovery without residual weakness
59 Y/F​ [19]​	Severe hyponatremia with optimal sodium correction	CPM	Pseudobulbar palsy, flaccid tetraplegia, ophthalmoplegia, hyper-reflexia	7 days	10 cycles 37300ml	Partial recovery with a mild residual tetraparesis Able to walk unaided, slight dysphonia and dysarthria persistent
^27 Y /M​ [^^6]^	Severe Hyponatremia with optimal correction	Mixed	Cogwheel rigidity, monotonous speech, spastic quadriparesis	N/A	5 cycles	Partial recovery – able to walk unaided with minimal residual tremor and rigidity.
24 Y/F​ [6]	Severe hyponatremia Hypokalemia Pregnant	Mixed	Spastic tetraperesis	N/A	5 cycles	Complete Recovery

## Conclusions

With this report, we conclude that ODS should be anticipated in severely hyponatremic patients even with optimal sodium correction. We highlight the potential role of plasmapheresis in treating ODS regardless of the duration of symptoms onset. Further research is needed to determine the efficacy of plasmapheresis in treating ODS. Nevertheless, this treatment option should be considered by healthcare providers for patients at risk of developing this condition.
